# Clinical and Sociodemographic Determinants of Adherence to World Cancer Research Fund/American Institute for Cancer Research (WCRF/AICR) Recommendations in Breast Cancer Survivors—Health-EpiGEICAM Study

**DOI:** 10.3390/cancers14194705

**Published:** 2022-09-27

**Authors:** Virginia Lope, Angel Guerrero-Zotano, Emma Ruiz-Moreno, Begoña Bermejo, Silvia Antolín, Álvaro Montaño, José Manuel Baena-Cañada, Manuel Ramos Vázquez, Nerea Fernández de Larrea-Baz, José Ignacio Chacón, José Angel García-Sáenz, Clara Olier, Montserrat Muñoz, Antonio Antón, Pedro Sánchez Rovira, Angels Arcusa Lanza, Sonia González, Amparo Oltra, Joan Brunet, Joaquín Gavilá Gregori, María Teresa Martínez, Lourdes Calvo, Libertad Rosell, Susana Bezares, Roberto Pastor-Barriuso, Beatriz Pérez-Gómez, Miguel Martín, Marina Pollán

**Affiliations:** 1 Department of Epidemiology of Chronic Diseases, National Center for Epidemiology, Instituto de Salud Carlos III, Avda. Monforte de Lemos 5, 28029 Madrid, Spain; 2 Consortium for Biomedical Research in Epidemiology & Public Health (CIBERESP), Instituto de Salud Carlos III, 28029 Madrid, Spain; 3 GEICAM Spanish Breast Cancer Group, Scientific Department, 28703 Madrid, Spain; 4 Medical Oncology, Instituto Valenciano Oncología, 46006 Valencia, Spain; 5 Medical Oncology, Hospital Clínico de Valencia, Biomedical Research Institute INCLIVA, 46010 Valencia, Spain; 6 Medical Unit, Centro de Investigación Biomédica en Red de Oncología, CIBERONC-ISCIII, 28029 Madrid, Spain; 7 Medicine Department, Universidad de Valencia, 46010 Valencia, Spain; 8 Medical Oncology, Complejo Hospitalario Universitario A Coruña -CHUAC, 15006 A Coruña, Spain; 9 Medical Oncology, Hospital Universitario Virgen del Rocío, 41013 Sevilla, Spain; 10 Medical Oncology, Hospital Puerta del Mar, Instituto de Investigación e Innovación Biomédica de Cádiz (INIBICA), 11009 Cádiz, Spain; 11 Medical Oncology, Centro Oncológico de Galicia, 15009 A Coruña, Spain; 12 Medical Oncology, Hospital Universitario de Toledo, 45007 Toledo, Spain; 13 Medical Oncology, Hospital Clínico San Carlos, IdISSC, 28040 Madrid, Spain; 14 Medical Oncology, Hospital de Alcorcón, 28922 Madrid, Spain; 15 Medical Oncology, Hospital Clinic i Provincial i IDIBAPS, 08036 Barcelona, Spain; 16 Medical Oncology, Hospital Universitario Miguel Servet, 50009 Zaragoza, Spain; 17 Medical Oncology, Hospital Universitario de Jaén, 23007 Jaén, Spain; 18 Medical Oncology, Consorci Sanitari de Terrassa, 08227 Terrassa, Spain; 19 Medical Oncology, Hospital Mutua Terrassa, 08221 Terrassa, Spain; 20 Medical Oncology, Hospital Virgen de los Lirios, 03804 Alicante, Spain; 21 Medical Oncology, Institut Català d’Oncologia, Hospital Josep Trueta, Oncogir, IDIBGI, 17007 Girona, Spain; 22 Medical Oncology, Instituto de Investigación Sanitaria Gregorio Marañón, 28007 Madrid, Spain

**Keywords:** lifestyle recommendations, WCRF/AICR guidelines, cancer prevention recommendations, compliance, health behaviours, healthy lifestyle, survival, breast cancer, Health-EpiGEICAM

## Abstract

**Simple Summary:**

There is insufficient evidence on the impact of sociodemographic and clinical factors on health-related lifestyles in breast cancer (BC) survivors. The International guidelines for cancer prevention are recommended not only for the general population but also for people who already have cancer. We sought to explore the degree of adherence to 2018 WCRF/AICR cancer prevention recommendations and to identify potentially influential factors, in 420 BC survivors in Spain. Global adherence was moderate, higher for the consumption of fruits and vegetables and lower for limiting red/processed meat consumption and having a high fibre intake. Compliance was worse in more educated survivors and in women with first-degree relatives with BC. There were differences in compliance with specific recommendations according to sociodemographic and clinical characteristics. This information is of interest to promote behavioural changes towards healthier lifestyles in BC survivors.

**Abstract:**

Breast cancer (BC) survivors are advised to follow the WCRF/AICR cancer prevention recommendations, given their high risk of developing a second tumour. We aimed to explore compliance with these recommendations in BC survivors and to identify potentially associated clinical and sociodemographic factors. A total of 420 BC survivors, aged 31–80, was recruited from 16 Spanish hospitals. Epidemiological, dietary and physical activity information was collected through questionnaires. A 7-item score to measure compliance with the recommendations was built according to the 2018 WCRF/AICR scoring criteria. Standardized prevalences and standardized prevalence ratios of moderate and high compliance across participant characteristics were estimated using multinomial and binary logistic regression models. The mean score was 3.9 (SD: 1.0) out of 7 points. Recommendations with the worst adherence were those of limiting consumption of red/processed meats (12% of compliance, 95% CI: 8.2–15.0) and high fibre intake (22% of compliance, 95% CI: 17.6–27.0), while the best compliance was observed for the consumption of fruits and vegetables (73% of compliance, 95% CI: 69.2–77.7). Overall, adherence was worse in women with university education and in those with first-degree relatives with BC. This information may be of interest to design and implement personalized preventive measures adapted to the characteristics of these patients.

## 1. Introduction

Breast cancer is the most frequently diagnosed tumour, both globally and in Europe [1]. In Spain, with an estimated age-standardized rate (2013 European standard population) of 132 cases per 100,000 in 2020, it represents 31% of all diagnosed cancer cases in women [2] and an increase of 10% is estimated for the year 2040 [3]. This tumour was also the leading cause of cancer mortality in women in 2019, with 6355 confirmed deaths (15% of all cancer deaths) [4]. The high incidence, together with the increase in survival, which attained a 5-year net survival of 85.5% in the period 2008–2013 [5], render this cancer the most prevalent in Spain, with an estimate of 516,827 prevalent cases in December 2020 (2140 cases/100,000) [6]. Increased survival entails a longer exposure time to health problems directly or indirectly related to BC, some of which are preventable by adopting healthy lifestyles.

In 2007, the World Cancer Research Fund/American Institute for Cancer Research (WCRF/AICR) issued 10 recommendations for cancer prevention related to diet, physical activity and weight management, based on the scientific evidence available at that time [7]. These recommendations were updated in 2018 [8,9]. Since the first report, numerous studies have associated adherence to these cancer prevention guidelines with lower BC risk [10,11,12,13,14]. The tenth recommendation addresses cancer survivors, advising them to follow the general recommendations for cancer prevention, if appropriate to their circumstances and unless otherwise advised by a health professional [9]. There is growing evidence that physical activity and other weight control measures (such as nutritional factors) may help to improve survival and health-related quality of life after BC diagnosis. Following these recommendations can also prevent new primary cancers or other non-communicable diseases [9,15]. Despite the increased number of BC survivors, there is insufficient evidence for the degree of knowledge and adherence to these recommendations and the impact of dietary and lifestyle interventions on them [16]. A review of 51 studies concluded that adherence to health behaviours included in the WCRF/AICR guidelines is low in cancer survivors, mainly among long-term survivors [17]. The quality of most published studies has been limited because they did not adequately account for relevant factors, such as cancer subtypes, type of treatment and other illnesses [9,16]. In addition, some studies have shown that certain sociodemographic factors (such as age, education, income level or marital status) are associated with adherence to cancer prevention recommendations in female cancer survivors [15,18,19]. The identification of these potentially influential factors can be useful to design specific strategies aimed at increasing adherence to evidence-based cancer prevention guidelines. Therefore, the present study sought to explore the degree of adherence to 2018 WCRF/AICR cancer prevention recommendations in BC survivors and to identify the clinical and sociodemographic conditions that influence such adherence.

## 2. Materials and Methods

### 2.1. Study Population

The EpiGEICAM study was a multicentre case-control study on incident female BC and individually matched controls carried out between 2006 and 2011. Histologically confirmed, in situ, and invasive BC cases, 18–70 years old, were recruited in the Oncology departments of 23 hospitals, members of the GEICAM Spanish Breast Cancer Group (https://www.geicam.org/, accessed on 26 September 2022) and located in 9 of the 17 Spanish Autonomous Regions. Cases living outside of the participant hospital areas, with previous history of BC, and those unable to answer the epidemiological questionnaire were excluded. Further details regarding the study design have been previously published [20,21].

In 2017, the Health-EpiGEICAM study was launched to assess changes in lifestyle and quality of life after diagnosis in the cohort of BC patients included in the EpiGEICAM study. Long-term BC survivors (mean time since diagnosis of 10 years, with a range between 7 and 12 years) were re-contacted and invited to participate in the new study by the Oncology units in which they had been diagnosed. Those who agreed to participate signed informed consent and received a series of questionnaires that they completed and returned to the GEICAM offices, where the information was recorded. In the Health-EpiGEICAM study, 16 of the 23 hospitals participated. Up to October 2019, of the 1017 BC cases who participated in the EpiGEICAM study, 767 could be re-contacted (75.4%).

Patient information was anonymized and de-identified prior to analysis. The protocol was conducted in accordance with the Declaration of Helsinki and the Ethics Committees of the 16 participant hospitals approved it.

### 2.2. Study Variables

Participants completed an epidemiological questionnaire, with demographic and anthropometric data, personal and family medical history, tobacco and physical exercise habits. At the time of recruitment, researchers from the hospitals completed another questionnaire with clinical and pathological information, including treatments received and disease evolution.

Dietary intake during the preceding year was estimated using the same 117-item semi-quantitative FFQ previously used in the EpiGEICAM study and validated in different Spanish adult populations [22]. The responses for each food item were converted to mean daily and weekly intake (in grams) and total energy intake (in kcals/day) based on the Food Composition Tables of Moreiras et al. [23]. Minutes of physical activity per week were calculated from a specific question in the epidemiological questionnaire that directly asked the specific activities carried out by the participants, the frequency (times per week), the duration (time spent in each session) and the time since starting. The total minutes/week for each participant was calculated by adding the minutes/weeks spent on each activity.

As possible determinants of adherence to the WCRF/AICR recommendations, we evaluated sociodemographic (age, educational level, region, marital status, employment situation, smoking habit and parity) and clinical factors (family BC history, number of comorbidities, menopausal status at diagnosis, years since diagnosis, BC subtype (hormone-receptor-positive tumours (HR+); human epidermal growth factor receptor 2 positive (HER2+) tumours; and triple-negative (TN) tumours), stage at diagnosis (according to the 7th edition of the American Joint Committee on Cancer (AJCC) TNM staging system) [24], previous and current cancer treatment and subsequent BC, including recurrent or second primary BC).

### 2.3. WCRF/AICR Score Construction

The components and scoring criteria to calculate the 2018 Standardized-WCRF/AICR score were based on the previously published guidelines by Shams-White et al. [25,26]. The score comprised eight components: (1) be a healthy weight, (2) be physically active, (3) eat a diet rich in wholegrains, vegetables, fruit and beans, (4) limit consumption of fast food and other processed foods high in fat, starches or sugar, (5) limit consumption of red and processed meat, (6) limit consumption of sugar-sweetened drinks, (7) limit alcohol consumption and (8) the optional inclusion of breastfeeding. We did not include the last recommendation due to lack of information. 

The method for estimating the score was based on the following criteria: 1 point was assigned when the recommendation was fully met, 0.5 points when the recommendation was partially met and 0 points when it was not met [25]. For the recommendation with two sub-recommendations (eat a diet rich in wholegrains, vegetables, fruit and beans) the scoring weight was divided equally between them to retain a total of one point. Be a healthy weight also had two sub-recommendations. However, since we did not have information on waist circumference, the body mass index (BMI) value was doubled as recommended [25]. An adapted ultra-processed foods (UPF) variable was created based on the NOVA classification system [25], which categorizes foods according to the extent and purpose of food processing, rather than in terms of nutrient composition [27]. Instead of recommending objective cut-points, the CUP expert panel recommended to group the UPF intake in tertiles within each specific dataset [25]. Physical activity cut-off points (min/wk) were based on Spanish national guidelines [28], as advised by the authors of the standard scoring system [25,26].

The final score was obtained as the sum of the individual scores. Each individual recommendation contributed equally to the total adherence score. Therefore, the overall score ranged from 0 (none of the recommendations were met) to 7 (all were fully met). The operationalizing, scoring and adherence to 2018 WCRF/AICR recommendations is presented in Table 1. According to the tertiles of the score distribution, three adherence categories were considered: low adherence (<3.50), moderate adherence (3.50–4.25) and high adherence (>4.25).

### 2.4. Statistical Analyses

Descriptive characteristics of participants were summarized using means and standard deviations for continuous variables and absolute figures and percentages for categorical variables. Significant differences by categories of the score were tested using Pearson chi-square for categorical variables and the Wald test, using simple linear regression models for continuous variables.

The prevalence of low, moderate and high compliance with 2018 WCRF/AICR cancer prevention recommendations by individual characteristics was standardized to the overall distribution of other sociodemographic and clinical characteristics in the entire sample of BC survivors. To this end, we fitted a multinomial logistic regression model adjusted for age, recruiting region, educational level, marital status (with or without partner), current job situation, smoking habit, energy intake, parity (parous or nulliparous), family history of BC, number of comorbidities, menopausal status at diagnosis, years since diagnosis, tumour subtype, AJCC stage at diagnosis and current cancer treatment. We then computed the averages of the predicted probabilities of low, moderate and high compliance, assuming that every participant was in each category of the individual characteristic [29,30]. The resulting estimates from this procedure represented the standardized prevalence of low, moderate and high compliance that would have been observed among women in a given factor category had their distribution of all other characteristics been equal to that of the entire sample of BC survivors, thus, controlling for confounding by direct standardization to the total sample. Standardized prevalence ratios (SPR) and 95% confidence intervals (95% CI) of moderate and high compliance were estimated across categories of each individual factor.

Using similar standardization methods based on binary logistic regression models, we also estimated standardized prevalences of high compliance with each recommendation, by sociodemographic and clinical characteristics of BC participants. These models were additionally adjusted for the overall distribution of the score obtained by adding all the individual recommendations except the one under study.

All analyses were performed using the statistical program STATA/MP 16.1 software (StataCorp LLC, College Station, TX, USA).

## 3. Results

After excluding 79 women who refused to participate, 202 with missing information in key clinical and/or epidemiological data, 13 with implausible energy intakes (<750 or >4500 kcal/day) and 53 women without information on one or more components of the score, the final Health-EpiGEICAM sample comprised 420 BC survivors, aged 31 to 80 years, with a mean follow-up of 10 years (9.95 ± 0.99; range: 7.4–12.4).

Table 1 shows the percentage of compliance with each WCRF/AICR recommendation. The one with the highest adherence was that of a high consumption of fruits and vegetables (73.6% consumed more than 400 g/day). On the contrary, the recommendation with the worst adherence was to limit the consumption of red and processed meat (10.7% of compliant women), followed by a high fibre intake (13.6% of compliant women).

**Table 1:** Operationalizing, scoring and adherence to 2018 WCRF/AICR recommendations in breast cancer survivors.

2018 WCRF/AICR Recommendations	Operationalization		Adherence
			(N = 420)
		Points	n (%)
1. Be a healthy weight	BMI (kg/m^2^):		
	18.5–24.9	1	192 (45.7)
	25–29.9	0.5	155 (36.9)
	<18.5 or >30	0	73 (17.4)
2. Be physically active	Total moderate-vigorous physical activity (min/wk):		
	>300	1	145 (34.5)
	150-<300	0.5	121 (28.8)
	<150	0	154 (36.7)
3. Eat a diet rich in wholegrains, vegetables, fruit and beans	Fruits and vegetables (g/day):		
	>400	0.5	309 (73.6)
	200-<400	0.25	85 (20.2)
	<200	0	26 (6.2)
	Total fibre (g/day):		
	>30	0.5	57 (13.6)
	15-<30	0.25	247 (58.8)
	<15	0	116 (27.6)
4. Limit consumption of “fast foods” and other processed foods high in fat, starches or sugars	Percent of total kcal from ultra-processed foods (aUPFs):		
	Tertile 1 (<14.0)	1	140 (33.3)
	Tertile 2 (14.0–20.9)	0.5	140 (33.3)
	Tertile 3 (>20.9)	0	140 (33.3)
5. Limit consumption of red and processed meat	Total red meat (g/wk) and processed meat (g/wk):		
	Red meat <500 and processed meat <21	1	45 (10.7)
	Red meat <500 and processed meat 21-<100	0.5	134 (31.9)
	Red meat >500 or processed meat >100	0	241 (57.4)
6. Limit consumption of sugar-sweetened drinks	Total sugar-sweetened drinks (g/day):		
	0	1	229 (54.5)
	>0-<250	0.5	176 (41.9)
	>250	0	15 (3.6)
7. Limit alcohol consumption	Total ethanol (g/day):		
	0	1	121 (28.8)
	<14 (1 drink)	0.5	260 (61.9)
	>14 (1 drink)	0	39 (9.3)

The distribution of sociodemographic and clinical characteristics is shown in Table 2. Mean age was 59 years (59.1 ± 9.0). A total of 38% of women had primary education or less and 30% university degree. Most of them had a partner (74%), were not active workers at the time of the interview (61%), did not smoke (81%) and had children (83%). The mean BMI was 26 Kg/m^2^ and the mean caloric intake 1775 Kcal/day (1775.0 ± 571.2). Regarding clinical factors, one in four women had more than three comorbidities. Nearly three-quarters (72%) of breast tumours were HR+ tumours and 11% TN. Stage III or IV were diagnosed in 12% of tumours. Under treatment (mainly hormonal treatment) were 14% of survivors and 12% had a recurrence or a second primary invasive BC. The overall score of adherence to the recommendations ranged between 1.25 and 7, with a mean of 3.9 ± 1.0. On average, high adherence was more prevalent in older women, in participants who did not work or smoke at diagnosis, among those with lower energy intake and in those with no family history of BC (Table 2).

Table 3 shows the SPR of moderate and high compliance with the WCRF/AICR cancer prevention recommendations by participant characteristics. The standardized prevalence of high compliance was 41% lower in women with university education compared with those with primary education or less (SPR: 0.59; 95% CI: 0.36–0.96) and 40% lower among women with first-degree relatives with BC compared to those with no family history (SPR: 0.60; 95% CI: 0.39–0.92). A sensitivity analysis excluding women who had recurrence, second primary invasive BC or who were undergoing treatment produced similar results (Supplementary Table S3).

**Figure 1:**
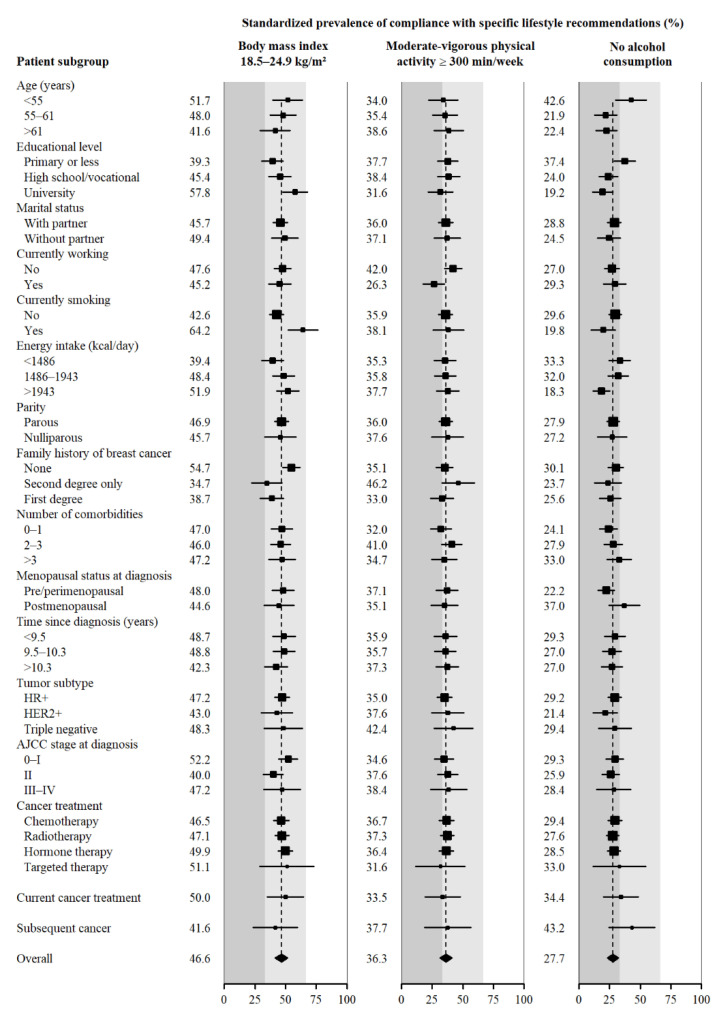
Standardised prevalence of high compliance with specific lifestyle recommendations by sociodemographic and clinical characteristics of breast cancer survivors (%). Standardised prevalence (95% CI) to the overall distribution of age, recruiting region, educational level, marital status, currently working, currently smoking, caloric intake, parity, family history of breast cancer, number of comorbidities, menopausal status at diagnosis, years since diagnosis, tumour subtype, AJCC stage at diagnosis, current cancer treatment and the overall adherence to the other recommendations in the entire sample of breast cancer survivors.

**Figure 2:**
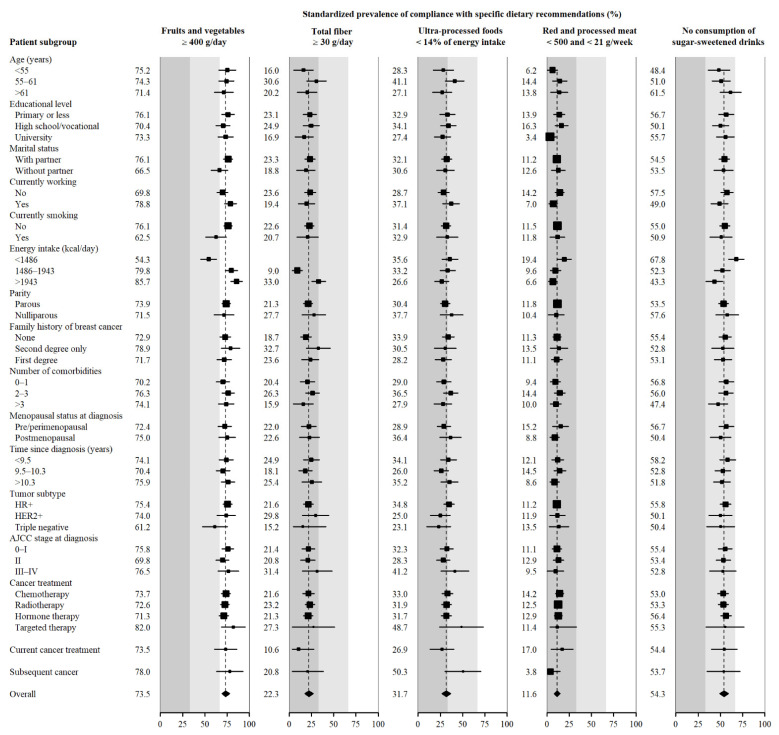
Standardised prevalence of high compliance with specific dietary recommendations by sociodemographic and clinical characteristics of breast cancer survivors (%). Standardised prevalence (95% CI) to the overall distribution of age, recruiting region, educational level, marital status, currently working, currently smoking, caloric intake, parity, family history of breast cancer, number of comorbidities, menopausal status at diagnosis, years since diagnosis, tumour subtype, AJCC stage at diagnosis, current cancer treatment and the overall adherence to the other recommendations in the entire sample of breast cancer survivors.

Figure 1 and Figure 2 show standardised prevalences of high compliance with each specific WCRF/AICR cancer prevention recommendation. The highest prevalence of compliance was observed for the fruits and vegetables intake recommendation (73.5% of compliance, 95% CI: 69.2–77.7), while the lowest adherence was observed for red and processed meat intake (11.6% of compliance, 95% CI: 8.2–15.0). Figure 1 and Supplementary Table S1 include recommendations related to BMI, physical activity and alcohol. Compliance with the recommendation of maintaining a healthy weight was higher among participants with a higher level of education, among current smokers, in those who did not have relatives with BC and in those who had been treated with hormone therapy. Regarding compliance with the physical activity recommendation, a higher prevalence of high compliance was observed in participants who did not work. Finally, the prevalence of teetotalers was higher among younger participants and among those with less education and lower among those who consumed more calories. 

Figure 2 and Supplementary Table S2 show Standardised prevalences of high compliance with the recommendations related to a healthy diet. Although the consumption of fruits, vegetables and fibre are included in the same recommendation, we decided to show them separately, given the huge difference in compliance between them. The prevalence of consumption of at least 400 g/day of fruits and vegetables was higher in women who did not smoke, in those with higher caloric intake and, to a lesser extent, among those who were working or had a stable partner. Adherence to the fibre intake recommendation was low, especially among patients with energy intake below the 66th percentile or patients who were under treatment. The prevalence of high adherence to the recommendation to limit red and processed meat consumption was higher in participants who were not working, in those with lower caloric intake and in those who had been treated with chemotherapy. In contrast, only 3% of university-educated participants and 4% of those with subsequent BC fully met this recommendation. Finally, the prevalence of not consuming sugar and sweetened drinks was higher in survivors with lower caloric intake.

## 4. Discussion

Although many studies have explored the association of the adherence to the WCRF/AICR cancer prevention guidelines with BC risk [10,11,12,13,14], fewer studies have analysed adherence to these recommendations with various cancer-related health outcomes among BC survivors, as reflected in the systematic review and meta-analysis published by Solans et al. [10]. Far fewer authors have explored the factors that influence compliance with cancer prevention recommendations among female cancer survivors [18,19]. The present study tries to evaluate the prevalence of healthy habits in BC survivors, taking as a reference the 2018 WCRF/AICR cancer prevention recommendations and the scoring criteria published by Shams-White et al. [25,26]. The study also tries to identify the sociodemographic and clinical factors that may be influencing adherence to healthier lifestyles. Our results show that global adherence to these recommendations is moderate, being higher for the recommendation related to fruits and vegetables consumption and lower for the recommendations to limit red and processed meat consumption and to maintain a diet rich in fibre. Overall adherence was lower in survivors with higher educational level and in women with first-degree relatives with BC. Our study also highlights differences in compliance with specific recommendations according to the characteristics of these women, which may be taken into account for tailoring preventive interventions or public health measures.

Tollosa et al. conducted a systematic review and meta-analysis on cancer survivors’ adherence to health behaviours related to WCRF/AICR recommendations, including 11 studies on BC survivors [17]. Although adherence to many behaviours was low, recent survivors reported relatively better adherence compared to long-term survivors, reflecting that the motivation to make healthy behaviour changes declines over time. This conclusion was later corroborated by the same authors in another follow-up study on long-term adherence to healthy behaviour recommendations in women diagnosed with BC [31]. Other authors evaluated adherence in African-American BC survivors, using the nutrition-related American guidelines for cancer prevention [18,32]. These studies showed a modest global adherence, being better in terms of meat intake and worse in terms of fruit and vegetable intake. Cross-sectional studies from a systematic review examining health behaviours and lifestyle interventions, also in African-American BC survivors [33], indicated poor adherence to physical activity and dietary intake and high rates of overweight and obesity. However, the 16 intervention studies included in that review indicated that survivors were able to make significant improvements in health behaviours, showing significant reductions in weight (range −1.9 to −3.6%), sedentary behaviour (−18%) and dietary fat intake (range −13 to −33%) and improvements in fruit and vegetable intake (range +25 to +55%) and physical activity (range +13 to +544%) [33]. Some sociodemographic factors associated with compliance with the WCRF/AICR recommendations described in our study could contribute to the design of interventions that motivate women to make long-lasting behavioural changes or to improve the effectiveness of ongoing interventions. In this sense, promoting physical activity in the workplace or making healthy dietary products cheaper to ensure greater access to them by women with less education could be good strategies.

The relative independence of each WCRF/AICR recommendation, both in terms of prevalence of adherence and associated factors, justifies the discussion of our results separately.

### 4.1. Be a Healthy Weight

Body fat is an important problem in many countries, including Spain, where 15.5% of women were obese and 30.6% overweight in 2020 [34]. Although weight gain is common in BC patients [35,36], prevalence of obesity and overweight in our sample (15% and 37%, respectively) are not very different from those estimated for the overall population in Spain. Less-educated participants, non-smokers and those with a family history of BC presented higher BMI. The inverse association with education has been consistently observed in general population [34]. Regarding cancer survivors, a recent study described that less-educated BC patients presented higher levels of inflammation than those with higher educational status, with BMI being a mediator of this association [37]. The lower body weight among smokers may be mediated by nicotine, which increases energy expenditure. However, smoking can also serve as a behavioural alternative to eating, resulting in decreased food intake [38]. Finally, with respect to family history, Harvie et al. also observed a significantly greater waist and waist/hip ratio among women with family BC history, possibly due to the genetic predisposition to central obesity of these women [39].

### 4.2. Be Physically Active

Multiple prospective observational studies have reported an association between lack of physical activity and BC recurrence and/or mortality [35,40] and there is also accumulating epidemiological evidence indicating that BC survivors participating in regular physical activity have reduced risk of cancer-related symptoms and improved physical health-related quality of life [35].

Cancer survivors tend to report less physical activity. The American Cancer Society’s Study of Cancer Survivors-II (ACS SCS-II) reported that 37% of women diagnosed with BC complied with the recommendation for physical activity published by this Society (at least 150 minutes of moderate-to-strenuous or 60 minutes of strenuous physical activity per week [41]). However, this figure varies depending on the time elapsed since diagnosis. A decline in physical activity is typically seen within 12 months of BC diagnosis. Although this rebounds in subsequent years, the increase does not return to pre-diagnosis levels [42]. In fact, the vast majority of BC survivors recruited in the study by Mason et al. [43] did not meet national exercise recommendations 10 years after diagnosis. In our study, also with a mean of 10 years post-diagnosis, 63% of women exceeded 150 min/week of moderate–vigorous physical activity. Participants who were working at the time of the interview were less compliant with this recommendation, probably due to lack of time.

### 4.3. Limit Alcohol Consumption

In agreement with other studies [18,44], more than 90% of our BC survivors were moderately or completely adherent to limiting alcohol. This is in line with the decrease in alcohol intake observed in Spain at the beginning of the 20th century and the stabilization in the last decade [45]. Prevalence of compliance with this recommendation was lower in women with higher caloric intake, in line with the findings of the meta-analysis by Kwok et al., in which it was observed that the consumption of alcoholic beverages significantly increased food energy intake and total energy intake [46]. Adherence was also lower in older survivors and in those with higher educational level. This pattern is also observed in the general population of women in Spain [34,47].

### 4.4. Eat a Diet Rich in Wholegrains, Vegetables, Fruit and Beans

Consistent with the 2018 WCRF/AICR standardised scoring system [25], we only operationalized the two goals pertaining to fruit and vegetable and total fibre intake.

Evidence on the role of fruit and vegetable intake in BC survivors is limited and it was not related to cancer survival in two similar meta-analyses [48,49]. Slightly stronger evidence exists regarding fibre intake, which, in the WCRF/AICR report, is classified as limited suggestive, linking increased consumption of foods containing fibre after diagnosis of primary BC with reduced risk of all-cause mortality [50]. Park et al., in a recent meta-analysis of cohort studies, observed that BC survivors with a dietary pattern enriched in vegetables, fruits and fibre had better prognosis [51]. Jayedi et al., in another dose–response meta-analysis of dietary fibre consumption and BC survival, reported a strong inverse linear association between dietary fibre intake and all-cause mortality risk [52].

Contrary to what was observed in the majority of BC survivors from several North-American studies [18,32,41], which failed to comply with the recommendation on the consumption of fruits and vegetables proposed by the American guidelines, the consumption of these foods was the dietary recommendation with the highest compliance in our participants. This is consistent with the high adherence to the Mediterranean diet in Spain. According to the last European Health Survey, 71.2% of Spanish women consumed fresh fruit daily in 2020 and 52% vegetables and salads [34]. However, despite the high consumption of fruits and vegetables by our participants, a low percentage reached the recommended daily intake of at least 30 g of total fibre. This low fibre intake has been previously described, both in BC survivors [53] and in the general Spanish population [54].

Women with higher caloric intake showed higher prevalence of compliance with this recommendation. The higher consumption of fruits and vegetables among non-smoking women is consistent with several previous studies, which found that never smokers reported more consumption than occasional and daily smokers [55,56].

### 4.5. Limit Consumption of “Fast Foods” and Other Processed Foods High in Fat, Starches or Sugars

According to a Food and Agriculture Organization (FAO) report based on household budget surveys, in just 20 years, from 1990 to 2010, the consumption of ultra-processed products tripled in Spain. In 2010, UPF accounted for 31.7% of daily caloric intake [27]. Further, in the Spanish population-based cohort of almost 12,000 individuals from the ENRICA study, average consumption of UPF represented 24.4% of the total energy intake [57]. In our study, this figure was somewhat lower (18%), although it must be taken into account that our participants are BC survivors and that red and processed meats and sugar-sweetened drinks have been excluded from this food group. Although we have not found information on UPF consumption in BC patients, Kenkhuis et al., in a study that evaluated the association of the 2018 WCRF/AICR dietary recommendations with patient-reported outcomes in colorectal cancer survivors, reported that the mean energy percentage from daily fast food intake among women was 35.2% [58]. The effect of this type of food on BC is unclear. In the French NutriNet-Santé cohort, a 10% increase in the proportion of UPF in the diet was associated with a significant 11% increase in BC risk [59], while Romagueda et al. found no evidence of association in the MCC-Spain case-control study [60].

### 4.6. Limit Consumption of Red and Processed Meat

This recommendation was the one that presented the worst adherence in our study participants, with more than half of these patients not complying. In the European Prospective Investigation into Cancer and Nutrition cohort, only 12% of BC cases and 17% of non-cases were fully compliant [61]. Very low adherence to this WCRF/AICR recommendation was also observed in colorectal cancer survivors from the Netherlands [58,62].

Despite the fact that Spain is still the EU country where the largest amount of meat is consumed [63], its intake decreased by 14% between 2012 and 2019, increasing again in 2020 as a result of the COVID-19 pandemic [64,65]. Although, in general, there is no conclusive evidence of the association between the consumption of red and processed meat with the incidence, recurrence or mortality due to BC [66,67,68], McCullough et al. detected, in BC survivors, an inverse association between post-diagnosis meat consumption and overall mortality [69].

In our study, only 3% of university graduates complied with the recommendation to limit meat consumption, far fewer than participants with lower education. This result is consistent with the pattern observed in the general population in Spain, where upper-middle class households consume 15% more meat than the national average, while lower social classes consume 11% less [65]. This could be related to the usually higher price of meat compared to other foods in Spain. On the other hand, the greater adherence in survivors who had been treated with chemotherapy is consistent with the results reported by de Vries et al. In this study, during chemotherapy, BC patients reported a significantly lower intake of meat products than women without cancer, probably due to secondary symptoms caused by this treatment, such as nausea, vomiting and loss of appetite [70]. In addition, meat aversion is commonly reported during chemotherapy [71].

### 4.7. Limit Consumption of Sugar-Sweetened Drinks

More than half of the participants in our study (54%) fully adhered to this recommendation and only 4% did not comply with it. These figures are much better than those reported by Karavasiloglou et al. among BC cases from the EPIC cohort (23% and 15%, respectively) [61] and somewhat better than those reported by BC cases from the Seguimiento Universidad de Navarra (SUN) cohort, where 40% never drank sugar-sweetened beverages [72]. However, the prevalence of consumption among our survivors is practically the same as the prevalence observed in women aged 55–64 years in the Spanish general population, where 3.9% consumed soft drinks with sugar daily and 55.1% never drank them in 2020 [34]. As expected, the prevalence of non-consumption of this type of product was higher in women with lower caloric intake.

### 4.8. Limitations and Strengths

To our knowledge, this is the first study that examined associations between adherence to the 2018 WCRF/AICR recommendations and clinical and sociodemographic factors in BC survivors in Spain. One of the strengths includes the method to estimate overall adherence to WCRF/AICR cancer prevention recommendations, based on the standard scoring system proposed by Shams-White et al. [25,26]. This method provides evidence-based cut-off points that facilitate the comparison of results with future studies. The mean 10-year follow-up (range: 7.4–12.4) of participants helps ensure that survivors’ behaviour was not influenced by treatments or possible lifestyle changes during the early stages of the disease. In addition, we exhaustively recorded clinical characteristics of our participants (e.g., staging, histological type or number of comorbidities), which allowed us to show estimates of the Standardised prevalence of adherence to the recommendations, controlled for these potential confounders, both for the general sample and for specific subgroups according to the participant characteristics.

This study also has a number of limitations that need to be considered. The first is related to the participation rate, since 10% of the patients refused to participate and 34% had incomplete data. This may have led to selection bias and may have affected the statistical power of our analyses. Second, the information on the participant characteristics was self-reported, so that behaviours considered socially desirable or healthy may have been overestimated, while other unhealthy behaviours may have been underestimated. Therefore, the global adherence to the WCRF/AICR recommendations might have been slightly overestimated. It is also possible that some information, mainly dietary assessment, could have been subject to recall bias. Although the FFQ used showed satisfactory reproducibility and validity [22], it is possible that the survivors had difficulties to fully and accurately recall their previous year intakes. Third, the lifestyle behaviours of BC survivors were assessed at a single time point, so we were unable to know whether adherence was a long-term practice or not. Fourth, survival bias may have occurred, since our study participants were women who survived BC and were healthy enough to participate and complete the questionnaires. This may have affected the external and internal validity of our results. When comparing baseline characteristics between women who participated in the Health-EpiGEICAM study and those who did not, we found that, at the time of diagnosis, women who participated were younger and had lower BMI. A higher percentage of them were university students, had a stable partner, one or no comorbidities, had less-aggressive tumours and less-advanced tumour stages than women who did not participate (Supplementary Table S4). Fifth, since many statistical comparisons were performed, we cannot rule out that one or more of them would be falsely positive. However, according to Professor Rothman [73], when studying biological associations, the premise that type I errors are the major concern may be wrong, so adjusting for multiple comparisons may not make sense. Sixth, we were limited by the small sample size when assessing factors associated with specific recommendations for cancer prevention. Finally, despite adjusting for a wide variety of potential confounders, residual confounding cannot be completely excluded.

## 5. Conclusions

In summary, this study highlights the low compliance with some international lifestyle recommendations for cancer prevention among BC survivors in Spain, such as high consumption of red and processed meat or low fibre intake. It also highlights the influence of certain sociodemographic and clinical factors on adherence to these recommendations. Knowledge of this information is of vital interest to integrate it at all levels of the health care system, including health promotion and public health services. It would help in the personalization of BC survivor care and in the design and implementation of preventive measures adapted to the characteristics of these patients. In addition, improving adherence to WCRF/AICR recommendations would have an impact not only on the prognosis of BC, but also on other pathologies that share risk factors with this tumour.

## Figures and Tables

**Table 2:** Sociodemographic and clinical characteristics of breast cancer survivors, overall and by tertiles of the 2018 WCRF/AICR score.

			Adherence to WCRF/AICR Recommendations			
		Total	Low	Moderate	High	
			(1.25–3.25)	(3.50–4.25)	(4.50–7)	
		n (%)	n (%)	n (%)	n (%)	*p*-value
Total		420 (100.0)	139 (33.1)	151 (36.0)	130 (31.0)	
Adherence score, mean (SD)		3.9 (1.0)				
Age, mean (SD)		59.1 (9.0)	57.6 (8.9)	59.5 (9.2)	60.2 (8.8)	0.014
Educational level						
	Primary education or less	156 (37.7)	46 (33.6)	56 (37.8)	54 (41.9)	0.016
	High school/vocational training	133 (32.1)	49 (35.8)	36 (24.3)	48 (37.2)	
	University graduate	125 (30.2)	42 (30.7)	56 (37.8)	27 (20.9)	
Marital status						
	With partner	308 (73.5)	99 (71.2)	113 (75.3)	96 (73.8)	0.727
	Without partner	111 (26.5)	40 (28.8)	37 (24.7)	34 (26.2)	
Currently working						
	No	250 (60.7)	70 (51.5)	92 (61.7)	88 (69.3)	0.012
	Yes	162 (39.3)	66 (48.5)	57 (38.3)	39 (30.7)	
Currently smoking						
	No	340 (81.3)	101 (73.2)	125 (82.8)	114 (88.4)	0.005
	Yes	78 (18.7)	37 (26.8)	26 (17.2)	15 (11.6)	
Energy intake kcal/d, mean (SD)		1775.0 (571.2)	1865.5 (628.8)	1746.0 (520.0)	1711.9 (555.4)	0.026
Parity						
	Parous	346 (82.8)	120 (86.3)	119 (79.3)	107 (82.9)	0.289
	Nulliparous	72 (17.2)	19 (13.7)	31 (20.7)	22 (17.1)	
Family history of breast cancer						
	None	227 (54.0)	73 (52.5)	71 (47.0)	83 (63.8)	0.062
	Second degree only	67 (16.0)	22 (15.8)	26 (17.2)	19 (14.6)	
	First degree	126 (30.0)	44 (31.7)	54 (35.8)	28 (21.5)	
Number of comorbidities						
	≤1	134 (35.4)	53 (41.7)	42 (30.9)	39 (33.6)	0.396
	2–3	152 (40.1)	44 (34.6)	58 (42.6)	50 (43.1)	
	>3	93 (24.5)	30 (23.6)	36 (26.5)	27 (23.3)	
Menopausal status at diagnosis						
	Pre/perimenopausal	240 (61.9)	85 (67.5)	84 (60.0)	71 (58.2)	0.276
	Postmenopausal	148 (38.1)	41 (32.5)	56 (40.0)	51 (41.8)	
Years since diagnosis		10.0 (1.0)	9.9 (0.9)	10.0 (1.0)	10.0 (1.0)	0.792
Tumour subtype ^a^						
	HR+	301 (71.7)	97 (69.8)	108 (71.5)	96 (73.8)	0.704
	HER2+	72 (17.1)	28 (20.1)	23 (15.2)	21 (16.2)	
	TN	47 (11.2)	14 (10.1)	20 (13.2)	13 (10.0)	
AJCC stage at diagnosis ^b^						
	0–I	189 (45.4)	57 (42.2)	74 (49.0)	58 (44.6)	0.290
	II	176 (42.3)	66 (48.9)	56 (37.1)	54 (41.5)	
	III–IV	51 (12.3)	12 (8.9)	21 (13.9)	18 (13.0)	
Cancer treatment						
	Chemotherapy	311 (74.4)	106 (76.8)	110 (72.8)	95 (73.6)	0.722
	Radiotherapy	326 (78.0)	106 (76.3)	114 (75.5)	106 (82.8)	0.283
	Hormonetherapy	340 (81.0)	111 (79.9)	121 (80.1)	108 (83.1)	0.758
	Targeted therapy	63 (15.0)	23 (16.5)	21 (13.9)	19 (14.6)	0.812
Current cancer treatment		57 (13.7)	24 (17.5)	17 (11.3)	16 (12.4)	0.267
Subsequent cancer ^c^		50 (11.9)	18 (13.0)	15 (9.9)	17 (13.1)	0.638

^a^ Tumour subtypes: HR+ = hormone receptor positive tumours (ooestrogen receptor, ER+ and/or progesterone receptor PR +, with HER2−); HER2+ = human epidermal growth factor receptor 2 positive tumours; TN = triple negative tumours (ER-, PR- and HER2-). ^b^ According to the 7th edition of the American Joint Committee on Cancer (AJCC) cancer staging manual [24]. ^c^ Recurrence or second primary invasive breast cancer.

**Table 3:** Standardized prevalence ratios of moderate and high compliance with 2018 WCRF/AICR recommendations by sociodemographic and clinical characteristics of breast cancer survivors.

		Standardized Prevalence Ratio (95% CI) ^a^	
		Moderate Compliance	High Compliance
Age, y ^b^			
	<55	1.00	1.00
	55–61	1.01 (0.67–1.53)	1.17 (0.79–1.74)
	>61	1.30 (0.74–2.28)	0.74 (0.39–1.41)
Educational level			
	Primary education or less	1.00	1.00
	High school/vocational training	0.89 (0.61–1.30)	0.97 (0.68–1.38)
	University graduate	1.39 (0.98–1.98)	0.59 (0.36–0.96)
Marital status			
	With partner	1.00	1.00
	Without partner	0.94 (0.67–1.34)	0.83 (0.55–1.26)
Currently working			
	No	1.00	1.00
	Yes	0.86 (0.60–1.24)	0.96 (0.65–1.41)
Currently smoking			
	No	1.00	1.00
	Yes	1.12 (0.78–1.61)	0.69 (0.42–1.16)
Energy intake, kcal/d ^b^			
	<1486	1.00	1.00
	1486–1943	1.22 (0.87–1.71)	0.81 (0.55–1.19)
	>1943	1.00 (0.68–1.46)	0.91 (0.62–1.33)
Parity			
	Parous	1.00	1.00
	Nulliparous	1.27 (0.90–1.80)	1.03 (0.67–1.59)
Family history of breast cancer			
	None	1.00	1.00
	Second degree only	1.12 (0.72–1.72)	0.79 (0.50–1.24)
	First degree	1.61 (1.19–2.19)	0.60 (0.39–0.92)
Number of comorbidities			
	≤1	1.00	1.00
	2–3	1.25 (0.89–1.77)	1.16 (0.81–1.68)
	>3	1.35 (0.92–1.99)	0.98 (0.63–1.53)
Menopausal status at diagnosis			
	Pre/perimenopausal	1.00	1.00
	Postmenopausal	0.90 (0.55–1.45)	1.38 (0.83–2.30)
Years since diagnosis ^b^			
	<9.53	1.00	1.00
	9.54–10.28	1.13 (0.79–1.60)	0.75 (0.50–1.14)
	>10.28	1.00 (0.68–1.48)	1.03 (0.70–1.53)
Tumour subtype ^c^			
	HR+	1.00	1.00
	HER2+	0.84 (0.54–1.30)	0.75 (0.47–1.22)
	TN	1.10 (0.71–1.71)	0.78 (0.44–1.39)
AJCC stage at diagnosis ^d^			
	0–I	1.00	1.00
	II	0.83 (0.60–1.15)	0.97 (0.68–1.40)
	III–IV	0.91 (0.57–1.44)	1.33 (0.85–2.09)
Cancer treatment			
	Chemotherapy	0.91 (0.63–1.31 )	1.14 (0.74–1.76)
	Radiotherapy	0.78 (0.57–1.07)	1.39 (0.89–2.20)
	Hormonetherapy	0.99 (0.63–1.57)	1.20 (0.69–2.08)
	Targeted therapy	1.29 (0.72–2.32)	1.12 (0.54–2.30)
Current cancer treatment		0.89 (0.56–1.41)	0.85 (0.50–1.44)
Subsequent cancer ^e^		1.01 (0.58–1.76)	1.06 (0.58–1.97)

^a^ Standardized to the overall distribution of age, recruiting region, educational level, marital status, currently working, currently smoking, caloric intake, parity, family history of breast cancer, number of comorbidities, menopausal status at diagnosis, years since diagnosis, tumour subtype, AJCC stage at diagnosis and current cancer treatment in the entire sample of breast cancer survivors. ^b^ In tertiles. ^c^ Tumour subtypes: HR+ = hormone receptor positive tumours (oestrogen receptor, ER+ and/or progesterone receptor PR +, with HER2−); HER2+ = human epidermal growth factor receptor 2 positive tumours; TN = triple negative tumours (ER-, PR- and HER2-). ^d^ According to the 7th edition of the American Joint Committee on Cancer (AJCC) cancer staging manual [24]. ^e^ Recurrence or second primary invasive breast cancer.

## Data Availability

The data presented in this study are available on request from the Principal Investigator. The data are not publicly available due to restrictions imposed by the Ethics Committees of the participating hospitals.

## Supplementary Materials

The following supporting information can be downloaded at: https://www.mdpi.com/article/10.3390/cancers14194705/s1, Table S1: Standardised prevalence of high compliance with specific lifestyle recommendations by sociodemographic and clinical characteristics of breast cancer survivors (%); Table S2: Standardised prevalence of high compliance with specific dietary recommendations by sociodemographic and clinical characteristics of breast cancer survivors (%); Table S3: Standardized prevalence ratios of moderate and high compliance with 2018 WCRF/AICR recommendations by sociodemographic and clinical characteristics of breast cancer survivors excluding women who have had a recurrence, second primary invasive breast cancer or are currently on treatment; Table S4: Baseline sociodemographic, lifestyle and clinical characteristics of the participants and non-participants in the Health-Epigeicam study.
