# Supplementary material for: Clinical and Sociodemographic Determinants of Adherence to World Cancer Research Fund/American Institute for Cancer Research (WCRF/AICR) Recommendations in Breast Cancer Survivors—Health-EpiGEICAM Study
Source: Cancers (Basel). 2022 Sep 27;14(19):4705. doi: 10.3390/cancers14194705 (PMC9561971; doi:10.3390/cancers14194705)
Supplement: Supplementary file 1 [file cancers-14-04705-s001.zip › cancers-1935939-supplementary.pdf]

Supplementary material

Clinical and sociodemographic determinants of adherence to World Cancer Research Fund/American Institute for Cancer Research (WCRF/AICR) recommendations in breast cancer survivors. Health-EpiGEICAM study.

Table S1. Standardized prevalence of high compliance with specific lifestyle recommendations by sociodemographic and clinical characteristics of breast cancer survivors (%).

	Body mass index (18.5-24.9 Kg/m^2)		Physical activity (≥ 300 min/week)		No alcohol consumption	
	Prev.[a]	(95%CI)	Prev.[a]	(95%CI)	Prev.[a]	(95%CI)
Overall	46.65	(41.59-51.70)	36.28	(31.31-41.25)	27.74	(23.26-32.23)
Age, y[b]						
<55	51.72	(39.54-63.91)	34.00	(21.94-46.07)	42.60	(29.98-55.23)
55-61	48.02	(37.52-58.52)	35.40	(25.23-45.57)	21.87	(12.78-30.95)
>61	41.59	(29.36-53.82)	38.58	(26.50-50.66)	22.38	(13.68-31.09)
P for homogeneity	*0.636*		*0.910*		*0.010*	
Educational level						
Primary education or less	39.30	(30.66-47.95)	37.72	(29.24-46.20)	37.35	(28.74-45.95)
High school / vocational training	45.43	(36.23-54.63)	38.40	(29.24-47.56)	23.98	(16.23-31.74)
University graduate	57.82	(47.49-68.15)	31.61	(21.38-41.84)	19.24	(10.91-27.57)
P for homogeneity	*0.038*		*0.594*		*0.016*	
Marital status						
With partner	45.70	(39.68-51.72)	35.99	(30.05-41.93)	28.84	(23.42-34.26)
Without partner	49.43	(38.51-60.35)	37.12	(26.34-47.90)	24.53	(15.21-33.86)
P for homogeneity	*0.572*		*0.863*		*0.452*	
Currently working						
No	47.57	(40.54-54.60)	42.04	(34.86-49.23)	26.96	(21.01-32.92)
Yes	45.18	(35.97-54.39)	26.28	(17.47-35.09)	29.26	(20.16-38.35)
P for homogeneity	*0.710*		*0.013*		*0.704*	
Currently smoking						
No	42.65	(36.93-48.37)	35.87	(30.30-41.44)	29.59	(24.42-34.76)
Yes	64.24	(51.99-76.49)	38.14	(25.48-50.79)	19.83	(9.96-29.70)
P for homogeneity	*0.002*		*0.754*		*0.093*	
Energy intake, kcal/d[b]						
<1486	39.38	(30.45-48.30)	35.34	(26.44-44.24)	33.33	(24.60-42.06)
1486-1943	48.42	(39.53-57.32)	35.78	(27.02-44.54)	32.00	(23.73-40.26)
>1943	51.89	(42.63-61.15)	37.69	(28.57-46.81)	18.34	(11.33-25.34)
P for homogeneity	*0.151*		*0.933*		*0.013*	
Parity						
Parous	46.85	(41.15-52.55)	36.01	(30.48-41.54)	27.85	(22.82-32.88)
Nulliparous	45.74	(33.01-58.46)	37.62	(24.53-50.71)	27.20	(15.10-39.31)
P for homogeneity	*0.879*		*0.828*		*0.925*	
Family history of breast cancer						
None	54.71	(47.62-61.80)	35.11	(28.25-41.97)	30.10	(23.70-36.49)
Second degree only	34.73	(22.35-47.12)	46.23	(32.80-59.66)	23.67	(12.56-34.77)
First degree	38.70	(29.19-48.21)	33.03	(23.76-42.31)	25.57	(16.88-34.26)
P for homogeneity	*0.004*		*0.271*		*0.541*	
Number of comorbidities						
≤1	47.02	(38.39-55.66)	31.98	(23.44-40.53)	24.14	(16.73-31.55)
2-3	45.96	(37.64-54.29)	41.00	(32.65-49.34)	27.89	(20.20-35.59)
>3	47.19	(36.24-58.13)	34.73	(24.23-45.24)	32.97	(23.00-42.95)
P for homogeneity	*0.980*		*0.329*		*0.398*	
Menopausal status at diagnosis						
Pre/perimenopausal	47.97	(39.17-56.77)	37.14	(28.21-46.07)	22.24	(15.35-29.12)
Postmenopausal	44.58	(32.38-56.79)	35.12	(24.07-46.16)	37.05	(24.54-49.56)
P for homogeneity	*0.717*		*0.819*		*0.089*	
Years since diagnosis[b]						
<9.53	48.71	(39.27-58.15)	35.90	(26.62-45.17)	29.33	(20.76-37.90)
9.54-10.28	48.76	(39.81-57.70)	35.68	(26.88-44.49)	26.96	(19.16-34.77)
>10.28	42.34	(32.84-51.83)	37.26	(27.92-46.60)	26.99	(18.42-35.56)

P for homogeneity	*0.581*		*0.970*		*0.910*	
Tumor subtype[c]						
HR+	47.25	(41.11-53.40)	35.04	(29.07-41.01)	29.18	(23.53-34.84)
HER2+	42.97	(30.13-55.80)	37.62	(24.51-50.74)	21.36	(11.27-31.44)
TN	48.34	(32.68-63.99)	42.35	(26.32-58.37)	29.37	(15.76-42.97)
P for homogeneity	*0.821*		*0.703*		*0.410*	
AJCC stage at diagnosis[d]						
0-I	52.22	(44.45-60.00)	34.61	(27.06-42.17)	29.33	(22.18-36.49)
II	39.97	(31.82-48.12)	37.56	(29.27-45.85)	25.86	(18.85-32.87)
III-IV	47.16	(32.16-62.17)	38.43	(23.63-53.22)	28.43	(14.42-42.44)
P for homogeneity	*0.121*		*0.849*		*0.800*	
Cancer treatment						
Chemotherapy	46.51	(40.16-52.85)	36.74	(30.53-42.96)	29.38	(23.55-35.21)
P for homogeneity[e]	*0.951*		*0.898*		*0.407*	
Radiotherapy	47.08	(41.34-52.81)	37.28	(31.62-42.94)	27.63	(22.50-32.76)
P for homogeneity[e]	*0.741*		*0.276*		*0.986*	
Hormonetherapy	49.89	(43.98-55.80)	36.38	(30.48-42.28)	28.53	(23.12-33.94)
P for homogeneity[e]	*0.031*		*0.950*		*0.587*	
Targeted therapy	51.10	(28.93-73.26)	31.63	(11.40-51.85)	33.04	(11.42-54.66)
P for homogeneity[e]	*0.686*		*0.645*		*0.619*	
Current cancer treatment	49.98	(35.14-64.83)	33.45	(18.98-47.91)	34.35	(20.17-48.53)
P for homogeneity[e]	*0.639*		*0.684*		*0.330*	
Subsequent cancer[f]	41.61	(23.52-59.70)	37.73	(18.76-56.70)	43.20	(24.56-61.83)
P for homogeneity[e]	*0.571*		*0.877*		*0.087*	

[a] Standardized to the overall distribution of age, recruiting region, educational level, marital status, currently working, currently smoking, caloric intake, parity, family history of breast cancer, number of comorbidities, menopausal status at diagnosis, years since diagnosis, tumor subtype, AJCC stage at diagnosis, current cancer treatment, and the overall adherence to the other recommendations in the entire sample of breast cancer survivors.

[b] In tertiles.

[c] Tumor subtypes: HR+= hormone receptor positive tumors (estrogen receptor, ER+ and/or progesterone receptor PR +, with HER2−); HER2+= human epidermal growth factor receptor 2 positive tumors; TN = triple negative tumors (ER-, PR-, and HER2-).

[d] According to the 7th edition of the American Joint Committee on Cancer (AJCC) cancer staging manual [24].

[e] P-value for homogeneity comparing with those who do not meet the condition.

[f] Recurrence or second primary invasive breast cancer.

Table S2. Standardized prevalence of high compliance with specific dietary recommendations by sociodemographic and clinical characteristics of breast cancer survivors (%).

	Fruits and vegetables (≥400 g/day)		Total fiber (≥30 g/day)		Ultra-processed foods (<14% of energy intake)		Red and processed meat (<500 and <21 g/week)		No consumption of sugar-sweetened drinks	
	Prev.[a]	(95%CI)	Prev.[a]	(95%CI)	Prev.[a]	(95%CI)	Prev.[a]	(95%CI)	Prev.[a]	(95%CI)
Overall	73.48	(69.24-77.71)	22.27	(17.57-26.98)	31.71	(26.92-36.50)	11.59	(8.21-14.97)	54.27	(49.28-59.26)
Age, y[b]										
<55	75.21	(65.65-84.77)	16.04	(5.06-27.02)	28.29	(16.84-39.75)	6.15	(0.56-11.75)	48.38	(36.18-60.59)
55-61	74.34	(65.73-82.96)	30.60	(19.63-41.58)	41.06	(30.56-51.56)	14.42	(6.37-22.48)	50.95	(40.77-61.13)
>61	71.38	(60.75-82.00)	20.19	(9.14-31.24)	27.08	(16.33-37.82)	13.82	(4.30-23.34)	61.48	(49.62-73.35)
P for homogeneity	*0.907*		*0.108*		*0.097*		*0.121*		*0.435*	
Educational level										
Primary education or less	76.14	(69.02-83.25)	23.10	(15.50-30.70)	32.91	(24.45-41.36)	13.90	(7.63-20.17)	56.69	(47.99-65.39)
High school / vocational training	70.37	(62.38-78.36)	24.92	(15.74-34.09)	34.15	(25.37-42.92)	16.30	(8.92-23.69)	50.14	(40.97-59.31)
University graduate	73.34	(64.64-82.03)	16.86	(6.75-26.98)	27.40	(18.09-36.71)	3.36	(1.06-10.14)	55.67	(45.57-65.77)
P for homogeneity	*0.579*		*0.508*		*0.570*		*0.001*		*0.563*	
Marital status										
With partner	76.14	(71.09-81.19)	23.31	(17.70-28.92)	32.12	(26.35-37.88)	11.24	(7.26-15.22)	54.53	(48.57-60.49)
Without partner	66.47	(56.91-76.03)	18.75	(8.37-29.13)	30.55	(20.51-40.59)	12.64	(4.90-20.38)	53.51	(42.79-64.23)
P for homogeneity	*0.094*		*0.466*		*0.799*		*0.763*		*0.876*	
Currently working										
No	69.78	(63.61-75.95)	23.64	(17.41-29.86)	28.68	(22.46-34.91)	14.22	(9.15-19.29)	57.45	(50.56-64.33)
Yes	78.78	(72.00-85.56)	19.37	(10.13-28.61)	37.13	(27.66-46.60)	7.01	(1.99-12.03)	49.01	(39.53-58.49)
P for homogeneity	*0.077*		*0.489*		*0.176*		*0.068*		*0.196*	
Currently smoking										
No	76.06	(71.34-80.77)	22.63	(17.25-28.01)	31.45	(26.08-36.81)	11.54	(7.69-15.38)	55.04	(49.42-60.66)
Yes	62.48	(50.76-74.20)	20.73	(8.77-32.69)	32.88	(20.82-44.93)	11.81	(3.55-20.07)	50.86	(38.38-63.33)
P for homogeneity	*0.040*		*0.785*		*0.835*		*0.955*		*0.557*	
Energy intake, kcal/d[b]										
<1486	54.26	(45.30-63.23)	0.00		35.65	(26.64-44.66)	19.43	(11.57-27.30)	67.85	(59.01-76.69)
1486-1943	79.75	(72.77-86.73)	9.01	(3.33-14.70)	33.23	(24.62-41.83)	9.63	(3.84-15.41)	52.28	(43.27-61.29)
>1943	85.65	(79.09-92.20)	32.99	(25.26-40.72)	26.63	(18.50-34.77)	6.63	(1.79-11.46)	43.26	(33.94-52.59)
P for homogeneity	*<0.001*		-		*0.334*		*0.028*		*0.001*	
Parity										
Parous	73.90	(69.13-78.68)	21.26	(16.13-26.38)	30.44	(25.12-35.76)	11.82	(8.03-15.61)	53.53	(47.88-59.17)
Nulliparous	71.54	(60.27-82.80)	27.70	(14.30-41.10)	37.67	(24.76-50.59)	10.38	(1.47-19.29)	57.63	(44.89-70.36)
P for homogeneity	*0.714*		*0.388*		*0.322*		*0.777*		*0.575*	
Family history of breast cancer										
None	72.86	(66.80-78.91)	18.69	(12.54-24.85)	33.85	(27.13-40.56)	11.35	(6.60-16.09)	55.40	(48.47-62.33)
Second degree only	78.93	(68.49-89.36)	32.71	(19.17-46.26)	30.54	(18.23-42.85)	13.50	(3.83-23.17)	52.76	(40.17-65.34)
First degree	71.66	(63.46-79.86)	23.65	(14.01-33.29)	28.22	(19.02-37.41)	11.09	(4.89-17.29)	53.06	(43.30-62.82)
P for homogeneity	*0.540*		*0.165*		*0.624*		*0.914*		*0.899*	

Number of comorbidities										
≤1	70.21	(62.58-77.85)	20.38	(12.17-28.60)	29.00	(20.87-37.12)	9.44	(3.67-15.21)	56.82	(48.29-65.35)
2-3	76.33	(69.38-83.29)	26.28	(18.42-34.14)	36.49	(28.34-44.65)	14.38	(8.31-20.45)	56.05	(47.90-64.20)
>3	74.07	(65.32-82.82)	15.88	(4.56-27.20)	27.93	(17.94-37.92)	9.99	(3.79-16.19)	47.43	(36.71-58.15)
P for homogeneity	*0.522*		*0.349*		*0.327*		*0.482*		*0.368*	
Menopausal status at diagnosis										
Pre/perimenopausal	72.40	(64.76-80.03)	22.03	(13.64-30.42)	28.93	(21.36-36.49)	15.24	(6.36-24.12)	56.73	(48.46-65.01)
Postmenopausal	75.00	(65.49-84.52)	22.63	(11.40-33.86)	36.38	(24.23-48.53)	8.76	(3.51-14.01)	50.41	(38.69-62.14)
P for homogeneity	*0.731*		*0.945*		*0.393*		*0.307*		*0.474*	
Years since diagnosis[c]										
<9.53	74.14	(66.20-82.08)	24.86	(15.84-33.88)	34.15	(25.18-43.11)	12.11	(5.35-18.87)	58.16	(49.21-67.12)
9.54-10.28	70.38	(62.65-78.12)	18.09	(10.50-25.68)	26.03	(17.89-34.17)	14.48	(7.78-21.19)	52.81	(43.96-61.66)
>10.28	75.92	(68.41-83.44)	25.36	(14.40-36.33)	35.21	(25.72-44.71)	8.55	(3.26-13.84)	51.77	(42.28-61.27)
P for homogeneity	*0.605*		*0.454*		*0.273*		*0.422*		*0.594*	
Tumor subtype[c]										
HR+	75.44	(70.32-80.55)	21.58	(16.14-27.02)	34.77	(28.80-40.74)	11.21	(7.09-15.32)	55.83	(49.82-61.83)
HER2+	74.02	(63.44-84.60)	29.78	(14.87-44.68)	25.00	(13.90-36.10)	11.86	(3.14-20.58)	50.10	(37.09-63.10)
TN	61.24	(47.23-75.26)	15.25	(4.41-41.22)	23.11	(9.68-36.54)	13.46	(2.33-24.59)	50.36	(34.54-66.18)
P for homogeneity	*0.183*		*0.440*		*0.162*		*0.935*		*0.665*	
AJCC stage at diagnosis[d]										
0-I	75.85	(69.41-82.29)	21.41	(14.33-28.49)	32.31	(24.96-39.66)	11.09	(5.84-16.34)	55.40	(47.73-63.08)
II	69.77	(62.46-77.07)	20.76	(12.56-28.96)	28.33	(20.75-35.90)	12.86	(6.90-18.83)	53.42	(45.27-61.58)
III-IV	76.46	(64.69-88.24)	31.41	(14.65-48.17)	41.24	(25.64-56.84)	9.54	(0.27-18.81)	52.78	(37.86-67.70)
P for homogeneity	*0.439*		*0.519*		*0.327*		*0.824*		*0.928*	
Cancer treatment										
Chemotherapy	73.66	(68.58-78.73)	21.58	(15.06-28.11)	32.97	(26.80-39.13)	14.21	(9.42-19.01)	53.05	(46.86-59.24)
P for homogeneity[e]	*0.811*		*0.856*		*0.465*		*0.018*		*0.394*	
Radiotherapy	72.63	(67.74-77.52)	23.23	(17.92-28.54)	31.87	(26.42-37.32)	12.52	(8.51-16.53)	53.27	(47.58-58.96)
P for homogeneity[e]	*0.558*		*0.277*		*0.830*		*0.349*		*0.596*	
Hormonetherapy	71.32	(66.00-76.64)	21.30	(16.11-26.49)	31.66	(26.07-37.25)	12.92	(8.42-17.42)	56.41	(50.63-62.19)
P for homogeneity[e]	*0.147*		*0.461*		*0.975*		*0.229*		*0.160*	
Targeted therapy	81.99	(68.43-95.55)	27.33	(3.53-51.14)	48.68	(24.12-73.25)	11.39	(3.21-33.23)	55.27	(33.85-76.69)
P for homogeneity[e]	*0.220*		*0.667*		*0.159*		*0.977*		*0.925*	
Current cancer treatment	73.48	(60.82-86.14)	10.62	(3.52-27.91)	26.86	(13.27-40.46)	17.03	(4.62-29.45)	54.42	(39.82-69.02)
P for homogeneity[e]	*0.999*		*0.038*		*0.461*		*0.356*		*0.983*	
Subsequent cancer[f]	78.02	(63.20-92.85)	20.79	(3.21-38.37)	50.33	(30.02-70.64)	3.82	(0.90-14.83)	53.73	(35.19-72.26)
P for homogeneity[e]	*0.536*		*0.864*		*0.060*		*0.013*		*0.952*	

[a] Standardized to the overall distribution of age, recruiting area, educational level, marital status, currently working, currently smoking, caloric intake, parity, family history of breast cancer, number of comorbidities, menopausal status at diagnosis, years since diagnosis, tumor subtype, AJCC stage at diagnosis, current cancer treatment, and the overall adherence to the other recommendations in the entire sample of breast cancer survivors.

[b] In tertiles.

[c] Tumor subtypes: HR+= hormone receptor positive tumors (estrogen receptor, ER+ and/or progesterone receptor PR +, with HER2−); HER2+= human epidermal growth factor receptor 2 positive

tumors; TN = triple negative tumors (ER-, PR-, and HER2-).
[d] According to the 7th edition of the American Joint Committee on Cancer (AJCC) cancer staging manual [24].
[e] P-value for homogeneity comparing with those who do not meet the condition.
[f] Recurrence or second primary invasive breast cancer.

Table S3. Standardized prevalence ratios of moderate and high compliance with 2018 WCRF/AICR recommendations by sociodemographic and clinical characteristics of breast cancer survivors excluding women who have had a recurrence, second primary invasive breast cancer or are currently on treatment (73 women excluded).

	Adjusted prevalence ratio (95%CI)[a]	
	Moderate compliance	High compliance
Age, y[b]		
<55	1.00	1.00
55-61	1.04 (0.66–1.64)	1.25 (0.80–1.97)
>61	1.41 (0.77–2.58)	0.89 (0.43–1.84)
Educational level		
Primary education or less	1.00	1.00
High school / vocational training	0.92 (0.62–1.38)	0.94 (0.64–1.38)
University graduate	1.38 (0.94–2.01)	0.64 (0.38–1.07)
Marital status		
With partner	1.00	1.00
Without partner	0.90 (0.61–1.32)	0.86 (0.55–1.34)
Currently working		
No	1.00	1.00
Yes	0.73 (0.48–1.10)	0.96 (0.63–1.47)
Currently smoking		
No	1.00	1.00
Yes	1.20 (0.82–1.77)	0.71 (0.41–1.24)
Energy intake, kcal/d[b]		
<1486	1.00	1.00
1486-1943	1.14 (0.78–1.67)	0.78 (0.52–1.18)
>1943	1.07 (0.72–1.60)	0.88 (0.58–1.32)
Parity		
Parous	1.00	1.00
Nulliparous	1.20 (0.80–1.81)	0.99 (0.60–1.62)
Family history of breast cancer		
None	1.00	1.00
Second degree only	1.19 (0.76–1.86)	0.78 (0.48–1.27)
First degree	1.55 (1.11–2.17)	0.63 (0.40–0.99)
Number of comorbidities		
≤1	1.00	1.00
2-3	1.17 (0.80–1.71)	1.04 (0.70–1.53)
>3	1.32 (0.86–2.01)	0.80 (0.49–1.30)
Menopausal status at diagnosis		
Pre/perimenopausal	1.00	1.00
Postmenopausal	0.81 (0.48–1.36)	1.20 (0.68–2.11)
Years since diagnosis[b]		
<9.53	1.00	1.00
9.54-10.28	1.01 (0.69–1.48)	0.88 (0.55–1.40)
>10.28	0.95 (0.64–1.42)	1.19 (0.78–1.82)
Tumor subtype[c]		
HR+	1.00	1.00
HER2+	0.81 (0.49–1.33)	0.86 (0.53–1.41)
TN	1.12 (0.71–1.77)	0.87 (0.49–1.55)
AJCC stage at diagnosis[d]		
0-I	1.00	1.00
II	0.88 (0.62–1.25)	0.98 (0.66–1.45)
III-IV	0.89 (0.52–1.54)	1.42 (0.88–2.29)
Cancer treatment		

Chemotherapy	0.93 (0.63-1.38)	1.02 (0.65-1.60)
Radiotherapy	0.78 (0.55-1.11)	1.66 (0.95-2.92)
Hormonetherapy	0.91 (0.56-1.47)	1.33 (0.73-2.39)
Targeted therapy	1.46 (0.80-2.68)	1.13 (0.51-2.51)

[a] Standardized to the overall distribution of age, recruiting region, educational level, marital status, currently working, currently smoking, caloric intake, parity, family history of breast cancer, number of comorbidities, menopausal status at diagnosis, years since diagnosis, tumor subtype, AJCC stage at diagnosis, and current cancer treatment in the entire sample of breast cancer survivors. [b] In tertiles. [c] Tumor subtypes: HR+= hormone receptor positive tumors (estrogen receptor, ER+ and/or progesterone receptor PR +, with HER2−); HER2+= human epidermal growth factor receptor 2 positive tumors; TN = triple negative tumors (ER-, PR-, and HER2-). [d] According to the 7th edition of the American Joint Committee on Cancer (AJCC) cancer staging manual [24].

Table S4. Baseline sociodemographic, lifestyle and clinical characteristics of the participants and non-participants in the Health-Epigeicam study.

Characteristics at diagnosis	Non-participants (N=347) n(%)	Participants (N=420) n(%)	*p*-value
Age, mean (SD)	52.2 (10.2)	49.3 (8.7)	<0.001
BMI (kg/m^2)	26.5 (5.0)	24.8 (4.1)	<0.001
Educational level			
Primary education or less	162 (54.2)	184 (43.8)	0.023
High school / vocational training	66 (22.1)	115 (27.4)	
University graduate	71 (23.7)	121 (28.8)	
Marital status			
With partner	218 (70.6)	304 (79.2)	0.009
Without partner	91 (29.4)	80 (20.8)	
Working status one year before diagnosis			
Not working	140 (46.4)	129 (33.7)	0.001
working	162 (53.6)	254 (66.3)	
Smoking status			
Non-smoker	240 (78.9)	305 (78.8)	0.965
Smoker	64 (21.1)	82 (21.2)	
Energy intake kcal/d, mean (SD)	1950.0 (614.6)	2023.7 (615.6)	0.139
Parity			
Parous	247 (79.7)	308 (79.6)	0.976
Nulliparous	63 (20.3)	79 (20.4)	
Family history of breast cancer			
None	237 (74.8)	292 (69.5)	0.172
Second degree only	41 (12.9)	56 (13.3)	
First degree	39 (12.3)	72 (17.1)	
Number of comorbidities			
≤1	239 (77.1)	335 (86.6)	<0.001
2-3	63 (20.3)	38 (9.8)	
>3	8 (2.6)	14 (3.6)	
Menopausal status			
Pre/perimenopausal	145 (46.8)	240 (61.9)	<0.001
Postmenopausal	165 (53.2)	148 (38.1)	
Tumor subtype[a]			
HR+	189 (61.2)	301 (71.7)	0.007
HER2+	80 (25.9)	72 (17.1)	
TN	40 (12.9)	47 (11.2)	
AJCC stage at diagnosis[b]			
0-I	100 (33.2)	189 (45.4)	<0.001
II	122 (40.5)	176 (42.3)	
III	59 (19.6)	49 (11.8)	
IV	20 (6.6)	2 (0.5)	

[a] Tumor subtypes: HR+= hormone receptor positive tumors (estrogen receptor, ER+ and/or progesterone receptor PR +, with HER2−); HER2+= human epidermal growth factor receptor 2 positive tumors; TN = triple negative tumors (ER-, PR-, and HER2-). [b] According to the 7th edition of the American Joint Committee on Cancer (AJCC) cancer staging manual [24]
